# The chain mediation effect of self-regulation and pre-sleep smartphone use on the relationship between smartphone dependence and bedtime procrastination among college students

**DOI:** 10.3389/fpubh.2026.1894257

**Published:** 2026-08-04

**Authors:** Wangqing Liu, Zhou Yuan, Shangfu Li, Yun Chen, Hangjiang Liu

**Affiliations:** 1 School of National Defense Education and Targeted Training, Jiangsu Maritime Institute, Nanjing, China; 2 School of Intelligent Control, Changzhou Institute of Industrial Technology, Changzhou, China; 3 Qiaoxing School of Economics and Management, Fujian Normal University of Technology, Fuzhou, China

**Keywords:** bedtime procrastination, chain mediation model, pre-sleep smartphone use, self-regulation, smartphone dependence

## Abstract

Smartphone dependence and bedtime procrastination are prevalent behavioral issues among college students, yet the psychological mechanisms linking them remain underexplored. This study investigated the chain mediating roles of self-regulation and pre-sleep smartphone use in the relationship between smartphone dependence and bedtime procrastination. A sample of 1,482 Chinese college students completed validated measures of smartphone dependence, self-regulation, pre-sleep smartphone use, and bedtime procrastination. Structural equation modeling and bootstrap analyses (5,000 resamples) were employed, controlling for gender and academic year. Results revealed that smartphone dependence positively predicted bedtime procrastination (standardized total effect of 0.56; direct effect of 0.21, *p* < 0.001). Both self-regulation and pre-sleep smartphone use served as significant independent mediators. More importantly, the serial mediation pathway—smartphone dependence → self-regulation → pre-sleep smartphone use → bedtime procrastination—was also significant, with a total indirect effect; the aggregate total indirect effect across all three mediation pathways was 0.35. Critically, the serial indirect pathway—smartphone dependence → self-regulation → pre-sleep smartphone use → bedtime procrastination—was statistically significant with a modest standardized coefficient of 0.07. Robustness checks supported the stability of the model. Multi-group analyses further showed that the path from smartphone dependence to pre-sleep smartphone use was stronger among female students than among male students (β = 0.55 vs. β = 0.42), whereas the effects of smartphone dependence on self-regulation (β = −0.35 vs. β = −0.15) and pre-sleep smartphone use (β = 0.60 vs. β = 0.40) were stronger among lower-grade students than among higher-grade students. These findings clarify the multi-path behavioral mechanism from smartphone dependence to bedtime procrastination, highlighting self-regulation and pre-sleep smartphone use as critical intervention targets. Limitations include cross-sectional design and self-report data, warranting future longitudinal and objective assessments.

## 1 Introduction

### 1.1 Research background

The rapid digitalization of society has rendered smartphones an indispensable component of college students' daily life, yet excessive and uncontrolled use has given rise to smartphone dependence a behavioral addiction characterized by compulsive use, loss of control, and impaired daily functioning (1). Recent studies have confirmed that smartphone dependence is closely associated with a series of negative outcomes among college students, including reduced academic engagement, emotional disorders, and disrupted sleep patterns (2, 3). Bedtime procrastination, defined as the voluntary delay of going to bed without external necessity, is a prevalent sleep-related behavioral problem in this population (4). Driven by irregular daily routines, elevated academic demands, and the ubiquitous presence of electronic devices, college students frequently exhibit delayed bedtime behaviors, which further lead to insufficient sleep duration, poor sleep quality, and even chronic sleep disorders (5).

A prominent behavioral consequence of smartphone dependence is bedtime procrastination the deliberate delay of bedtime despite awareness of negative outcomes and no external obligations. This phenomenon affects over 60% of college students, leading to shortened sleep duration, diminished sleep quality, daytime fatigue, impaired academic performance, and elevated risks of anxiety and depression (6, 7). Pre-sleep smartphone use (e.g., scrolling social media or viewing videos in bed) exacerbates this issue by displacing sleep time while delivering immediate rewards that override long-term self-regulatory goals. Extensive literature has established a robust positive association between smartphone dependence and bedtime procrastination. Yet, the underlying psychological and behavioral mechanisms, particularly involving self-regulation and pre-sleep smartphone use, remain incompletely elucidated. Self-regulation, encompassing monitoring, planning, impulse inhibition, and emotional regulation, serves as a critical resource depleted by chronic smartphone engagement, according to Resource Depletion Theory and Dual-Process Theory of Self-Regulation (8).

Empirical evidence consistently supports these pathways. Longitudinal studies further reveal bidirectional relations: problematic smartphone use predicts subsequent bedtime procrastination, while bedtime procrastination reciprocally intensifies smartphone dependence, with self-control acting as a key mediator (9). Pre-sleep smartphone use behaviors (duration, frequency, and content) function as a proximal behavioral bridge, with excessive engagement reinforcing immediate gratification over sleep intentions (10).

Additional research highlights mediating and moderating factors. For instance, physical activity and anxiety exhibit masking or chain-mediating effects on the link between mobile phone addiction and bedtime procrastination (11). Gender and academic year differences emerge, with females and lower-year students often displaying stronger associations due to heightened social media engagement and transitional stressors. Latent profile analyses identify subgroups of bedtime procrastination among Chinese college students, influenced by problematic smartphone use, depression, anxiety, and self-control as protective or risk factors (12).

Although previous studies have consistently linked smartphone dependence to bedtime procrastination, the mechanisms explaining this association remain fragmented (13). Some studies have emphasized the role of diminished self-control or self-regulatory resources, whereas others have focused on bedtime smartphone engagement as a proximal behavioral factor (14, 15). Pre-sleep smartphone use, as a specific behavioral link, directly delays bedtime and disrupts sleep initiation through physiological, psychological, and behavioral pathways (16). However, these two explanations have often been examined separately, leaving unclear how a relatively stable dependence tendency is translated into concrete pre-sleep behaviors and, subsequently, delayed bedtime. This gap is theoretically important because bedtime procrastination is not merely a consequence of high smartphone involvement; rather, it may emerge through a sequential process in which dependence weakens self-regulatory capacity, reduced self-regulation increases susceptibility to pre-sleep smartphone use, and prolonged pre-sleep use finally displaces intended bedtime. Drawing on the research frameworks of Tang et al. (4) and Guo (17) on chain mediation, this study supplements the theoretical and measurement content of self-regulation, constructs a visual chain mediation model, and empirically tests the multi-path transmission mechanism from smartphone dependence to bedtime procrastination, with the aim of furnishing a more comprehensive theoretical account and a robust foundation for targeted interventions.

### 1.2 Theoretical rationale and research hypotheses

#### 1.2.1 . Theoretical rationale

This study is grounded in Resource Depletion Theory and the Dual Process Theory of Self-Regulation to construct and test a serial mediation model linking smartphone dependence to bedtime procrastination via pre-sleep smartphone use. Resource Depletion Theory posits that self-regulatory resources are finite and that persistent smartphone use depletes these resources, impairing impulse control and time management (18), thereby directly increasing the frequency and duration of pre-sleep smartphone use and promoting bedtime procrastination (19). The Dual Process Theory further explains individual differences: among college students with high smartphone dependence, the impulsive system (seeking immediate gratification) tends to override the reflective system (rational planning), while the weakened reflective system—manifested as low self-regulation—fails to effectively curb the urge for pre-sleep use (20). Within this theoretical chain, self-regulation and its four sub-mechanisms—monitoring, planning, impulse inhibition, and emotion regulation (21)—constitute the core mediating link. Specifically, smartphone dependence impairs monitoring (failure to perceive excessive pre-bed use), undermines planning (inability to establish regular sleep routines), weakens impulse inhibition (difficulty resisting notifications and social feedback), and disrupts emotion regulation (using phones to escape anxiety and boredom), ultimately exacerbating bedtime procrastination (22–24).

Extensive empirical evidence supports this serial pathway: chronic smartphone engagement depletes self-control resources (25, 26), and self-control serves as a key mediator between smartphone dependence and bedtime procrastination (27); when resources are exhausted, the impulsive system dominates behavioral decisions (28, 29). Moreover, pre-sleep smartphone use is a proximal behavioral outcome of self-regulatory depletion (30, 31), together forming the complete mediation chain. This effect is further moderated by gender, academic year, academic stress, and physical activity levels (32). Meta-analytic syntheses confirm a robust association between smartphone addiction and various forms of procrastination, underscoring the need for integrated chain models that incorporate both psychological (self-regulation) and behavioral (pre-sleep use) mediators (33).

#### 1.2.2 . Research hypotheses

Based on the above theoretical analysis and a comprehensive review of existing literature, the following research hypotheses are proposed:

H1: Smartphone dependence is positively associated with bedtime procrastination among college students.

H2: Self-regulation statistically mediates the association between smartphone dependence and bedtime procrastination.

H3: Pre-sleep smartphone use statistically mediates the association between smartphone dependence and bedtime procrastination.

H4: Self-regulation and pre-sleep smartphone use form a significant serial indirect pathway linking smartphone dependence and bedtime procrastination.

### 1.3 Research significance

#### 1.3.1 . Theoretical significance

First, this study enriches the theoretical connotation and measurement basis of self-regulation in the context of smartphone dependence and bedtime procrastination. By clarifying the four core sub-mechanisms of self-regulation and their specific role in the behavioral transmission process, enriching the research on self-regulation theory in the field of digital age sleep behavior. Second, by constructing a visualized chain mediation model and empirically testing the sequential mediating role of self-regulation and pre-sleep smartphone use, this study reveals the multi-path transmission mechanism from smartphone dependence to bedtime procrastination, filling the research gap in the joint action of psychological and behavioral factors in this relationship. Third, the study verifies the applicability of Resource Depletion Theory and the Dual Process Theory of Self-Regulation to college students' smartphone use and sleep behavior, expanding the application scope of these theories in the digital context.

#### 1.3.2 . Practical significance

The research results provide clear and actionable intervention strategies for colleges and universities to address college students' smartphone dependence and bedtime procrastination. By identifying self-regulation and pre-sleep smartphone use as key intervention nodes, the study can guide colleges to design targeted intervention programs, such as self-regulation training courses (focusing on impulse inhibition and time management) and pre-sleep digital detox activities. In addition, the heterogeneity findings offer a basis for personalized intervention. For female students and lower-grade students, targeted strategies can be formulated to improve self-regulation ability and regulate pre-sleep smartphone use behavior. The visualized chain mediation model also helps educators and psychological counselors more intuitively understand the internal mechanism of the problem and formulate more targeted intervention plans.

## 2 Methods

### 2.1 Participants

A stratified cluster sampling method was used to recruit college students from three universities (comprehensive, science and engineering, and normal universities) in China. A total of 1,550 online questionnaires were distributed, and invalid questionnaires were excluded according to preset quality control standards (e.g., excessively short completion time, failure to pass attention-check items, repetitive responses, and obviously invalid information). Finally, 1,482 valid questionnaires were obtained, with an effective recovery rate of 95.61%. Among the participants, 612 (41.3%) were male, 850 (57.3%) were female, and 20 (1.4%) did not specify their gender; 380 (25.6%) were freshmen, 360 (24.3%) were sophomores, 380 (25.6%) were juniors, and 362 (24.4%) were seniors; the average age was 20.4 ± 1.6 years old. All participants signed an electronic informed consent form before completing the questionnaire, and the study was approved by the Institutional Review Board of the corresponding university, in compliance with the ethical standards of psychological research.

A stratified cluster sampling procedure was adopted. First, universities were stratified by institutional type, including one comprehensive university, one science-and-engineering university, and one normal university. Within each university, intact classes were selected from each academic year to ensure approximate coverage of freshmen, sophomores, juniors, and seniors. All students in the selected classes were invited to participate through class-based online survey links. The inclusion criteria were: as follows (a) being a full-time undergraduate student, (b) having used a smartphone for at least 6 months. (c) Providing informed consent. The exclusion criteria were:as follows (a) incomplete questionnaires. (b) Completion time shorter than one-third of the median completion time, (c) failure on at least one attention-check item, (d) invariant responses across long item blocks, and (e) logically inconsistent demographic information.

### 2.2 Measures

#### 2.2.1 . Smartphone dependence

All scale adaptation and validation procedures followed a standardized process. For scales originally developed in English, translation and back-translation were conducted by two bilingual researchers. Discrepancies were resolved through discussion with a third researcher. For revised, shortened, or study-specific measures, item selection and wording revisions were based on theoretical relevance, previous literature, and expert review. Three experts in psychology and behavioral science evaluated item clarity, content relevance, and construct representativeness. A pilot test was conducted with [120] college students who were not included in the final sample. The final item list, scoring rules, standardized factor loadings, composite reliability, average variance extracted, and inter-construct correlations are provided in Supplementary Table S1.

The Smartphone Addiction Scale-Short Version (SAS-SV) (34) was adapted and used to measure smartphone dependence, with a total of 8 items covering three dimensions: use impulsivity, loss of control, and functional impairment. The scale uses a 5-point Likert rating (1 = completely disagree, 5 = completely agree), with higher total scores indicating a higher degree of smartphone dependence. A sample item is “I often cannot reduce my smartphone use when I want to”. In this study, the Cronbach's α coefficient of the scale was 0.86, and the confirmatory factor analysis (CFA) fitting indices were good (χ/*df* = 2.30, CFI = 0.95, TLI = 0.93, RMSEA = 0.058, SRMR = 0.042), indicating good reliability and construct validity.

#### 2.2.2 . Self-regulation

The Self-Regulation Questionnaire (SRQ) (21) was revised and optimized for the college student population, based on the four core sub-mechanisms of self-regulation (monitoring, planning, impulse inhibition, and emotional regulation). The scale consists of 10 items, with 2–3 items for each sub-mechanism, designed to comprehensively assess the overall self-regulation ability of college students. The scale uses a 5-point Likert rating (1 = completely disagree, 5 = completely agree), with higher scores indicating stronger self-regulation ability. Monitoring dimension example: “I can timely notice when I spend too much time on my phone before going to bed;” Planning dimension example: “I can set a fixed time to stop using my phone before going to bed and stick to it;” Impulse inhibition dimension example: “I can resist the temptation to check my phone when I receive notifications before going to bed;” Emotional regulation dimension example: “I will not use my phone to escape negative emotions such as anxiety and boredom before going to bed”.

In this study, the Cronbach's α coefficient of the revised SRQ was 0.88, indicating good internal consistency reliability. CFA results showed that the four-factor model of the scale had good fit with the research data (χ/*df* = 2.10, CFI = 0.96, TLI = 0.95, RMSEA = 0.052, SRMR = 0.039). Convergent validity was verified by the average variance extracted (AVE) of each dimension (0.52–0.61, all > 0.50) and composite reliability (CR) (0.78–0.85, all > 0.70); discriminant validity was verified by the square root of AVE of each dimension being greater than the correlation coefficient between the dimension and other dimensions, demonstrating good construct validity. The revised Self-Regulation Questionnaire was adapted from Tangney et al.'s self-control framework and revised to fit bedtime-related smartphone-use contexts among Chinese college students. Items were selected to represent four theoretically defined subdimensions: monitoring, planning, impulse inhibition, and emotional regulation. The wording of several items was modified to reflect pre-sleep behavioral contexts, but the core meaning of self-regulatory capacity was retained. Each item was rated on a 5-point Likert scale, with higher scores indicating stronger self-regulation. Standardized factor loadings ranged from 0.53 to 0.84. Composite reliability values ranged from 0.78 to 0.85, and AVE values ranged from 0.52 to 0.61. No residual correlations were added unless theoretically justified and reported in Supplementary Table S1.

#### 2.2.3 Pre-sleep smartphone use

Pre-sleep smartphone use was measured using a study-specific index developed to capture bedtime-related smartphone behaviors that are directly relevant to the present chain mediation model. Existing problematic smartphone use scales mainly assess general dependence symptoms, functional impairment, or loss of control, whereas the present study required a more proximal measure of smartphone behavior immediately before bedtime. Therefore, items were designed to assess three behavioral aspects: use duration, use frequency, and use purpose within the pre-sleep period. The index included 6 items. Duration and frequency items were scored from 1 to 5 after categorical grading, and use-purpose items were rated on a 5-point Likert scale. Higher scores indicated more frequent and prolonged pre-sleep smartphone use.

Although this index showed acceptable internal consistency in the present sample (Cronbach's α = 0.81) and acceptable CFA fit (χ/*df* = 2.70, CFI = 0.94, TLI = 0.92, RMSEA = 0.063, SRMR = 0.048), it should be regarded as a context-specific behavioral measure rather than a fully validated independent diagnostic scale.

#### 2.2.4 . Bedtime procrastination

The Bedtime Procrastination Scale (BPS) developed by Kroese et al. (4) was shortened and adapted, with a total of 9 items measuring the frequency and degree of voluntary bedtime delay among college students. The scale uses a 5-point Likert rating (1 = completely disagree, 5 = completely agree), with higher total scores indicating more severe bedtime procrastination. Example items include “I often find myself going to bed later than planned” and “Even if I know I need to get up early the next day, I still delay going to bed to use my phone”. In this study, the Cronbach's α coefficient of the scale was 0.90, and the CFA fitting indices were χ/*df* = 1.90, CFI = 0.97, TLI = 0.96, RMSEA = 0.045, SRMR = 0.035, indicating excellent reliability and construct validity. The shortened Bedtime Procrastination Scale was adapted from the original Bedtime Procrastination Scale to reduce participant burden and improve contextual relevance for Chinese college students. Items were selected based on theoretical representativeness and factor loading performance in previous studies. To reduce construct overlap with smartphone-related variables, items explicitly referring to smartphone use were examined in a sensitivity analysis. All items were rated on a 5-point Likert scale, with higher scores indicating more severe bedtime procrastination. Standardized factor loadings ranged from 0.57 to 0.81, CR was [0.83], and AVE was 0.54. The complete shortened scale is reported in Supplementary Table S1. Depression symptoms were measured using the Patient Health Questionnaire-9 (PHQ-9). The scale includes nine items assessing depressive symptoms over the past 2 weeks. Each item is rated from 0 to 3, with higher scores indicating more severe depressive symptoms. In the present sample, Cronbach's α was 0.82. Anxiety symptoms were measured using the Generalized Anxiety Disorder-7 scale (GAD-7), which includes seven items rated from 0 to 3. Higher scores indicate more severe anxiety symptoms. In the present sample, Cronbach's α was 0.84. Depression and anxiety were included as robustness covariates because previous studies have linked mental health symptoms to both problematic smartphone use and sleep-related procrastination, making them plausible confounding variables in the association between smartphone dependence and bedtime procrastination.

### 2.3 Data collection procedure

Data quality was controlled using pre-specified criteria. Of the 1,550 submitted questionnaires, 68 were excluded before analysis. Specifically, questionnaires were removed if they met any of the following criteria: incomplete responses (*n* = 28), completion time shorter than 3 min or shorter than one-third of the sample median completion time (*n* = 16), failure to correctly answer at least one of the three attention-check items (*n* = 12), invariant or highly repetitive responses across more than [xx] % of scale items (*n* = 8), or logically inconsistent demographic information (*n* = 4). When a questionnaire met more than one exclusion criterion, it was classified according to the first criterion detected in the cleaning sequence. The final analytic sample included 1,482 valid responses, yielding an effective response rate of 95.61%. The questionnaires were distributed through a professional online questionnaire platform, and recruitment information was released through college class groups, student organizations, and official campus announcements. The survey was conducted during the fall semester of the academic year, and participants completed the questionnaire anonymously, with an average completion time of about 15 min. The questionnaire included an informed consent statement, four standardized scales (as described in 2.2), basic demographic information (gender, age, grade, major, etc.), and three attention-check questions (e.g., “Please select ‘completely agree' for this item”) to ensure the quality of the collected data. After the data collection was completed, the raw data were sorted and cleaned, and invalid questionnaires were excluded according to preset standards to form the final research sample. Of the 1,550 submitted questionnaires, 68 were excluded before analysis. Specifically, 28 were excluded because of incomplete responses, 16 because of excessively short completion time, 12 because of failed attention-check items, 8 because of invariant or repetitive response patterns, and 4 because of logically inconsistent information. When a questionnaire met multiple exclusion criteria, it was classified according to the first criterion detected in the data-cleaning sequence.

Several procedural remedies were adopted to reduce potential common method bias. First, the survey was anonymous, and participants were informed that there were no right or wrong answers. Second, individual responses were used only for research purposes and were not disclosed to instructors or administrators. Third, attention-check items were included to identify careless responses. Fourth, the measures were presented in separate sections with clear instructions to reduce respondents' tendency to answer all items in the same manner.

### 2.4 Data analysis

SPSS 26.0 and AMOS 24.0 software were used for statistical analysis of the data, and the specific analysis steps were as follows:

1. Descriptive statistics and correlation analysis: Means, standard deviations, skewness, and kurtosis were calculated for each main variable to assess normality. Pearson correlation analysis was conducted to examine bivariate relationships among smartphone dependence, self-regulation, pre-sleep smartphone use, and bedtime procrastination.
2. Reliability and validity test: Internal consistency reliability of each scale was assessed using Cronbach's α coefficient. CFA was performed to evaluate construct validity, including convergent and discriminant validity.
3. Common method bias was assessed using multiple approaches. First, Harman's single-factor test was conducted as a preliminary diagnostic. Second, a common latent factor model was estimated by adding an unmeasured latent factor loading on all measurement items. The standardized path coefficients before and after adding the common latent factor were compared to evaluate whether the main findings were substantially affected by common method variance.
4. Chain mediation model test: A structural equation model was constructed with gender and academic year as control variables. The significance of direct and indirect effects was tested using the bootstrap method with 5,000 resamples. A 95% confidence interval (CI) was used to determine the significance of mediating effects; an effect was considered statistically significant if the CI did not include zero.
5. Robustness analyses were conducted in two ways. First, the composite measure of pre-sleep smartphone use was replaced with three single-dimension indicators: use duration within 1 h before bedtime, use frequency before bedtime, and use purpose before bedtime. The duration indicator was selected as the primary alternative indicator because it directly reflects sleep-time displacement, which is theoretically closest to bedtime procrastination. Frequency and purpose indicators were additionally tested to examine whether the findings were stable across different operational definitions of pre-sleep smartphone use. Second, depression and anxiety were added as additional covariates.
6. Multi-group SEM was conducted separately for gender and academic-year groups. For gender comparisons, only participants who reported male or female gender were included in the multi-group analysis; the 20 participants who did not report gender were retained in the main full-sample analysis by using a separate missing/unspecified gender category or were excluded from gender-specific multi-group analysis only. For academic-year comparisons, freshmen and sophomores were classified as lower-grade students, whereas juniors and seniors were classified as higher-grade students. This grouping was based on the distinction between early college adaptation and later-stage academic development. Measurement invariance was tested separately for gender and academic-year groups, including configural, metric, and scalar invariance. Between-group differences in structural paths were examined by comparing unconstrained models with models in which each focal path was constrained to be equal across groups. A significant chi-square difference test or Wald test was used as evidence of a statistically significant group difference.

### 2.5 Chain mediation model construction

**Figure 1:**
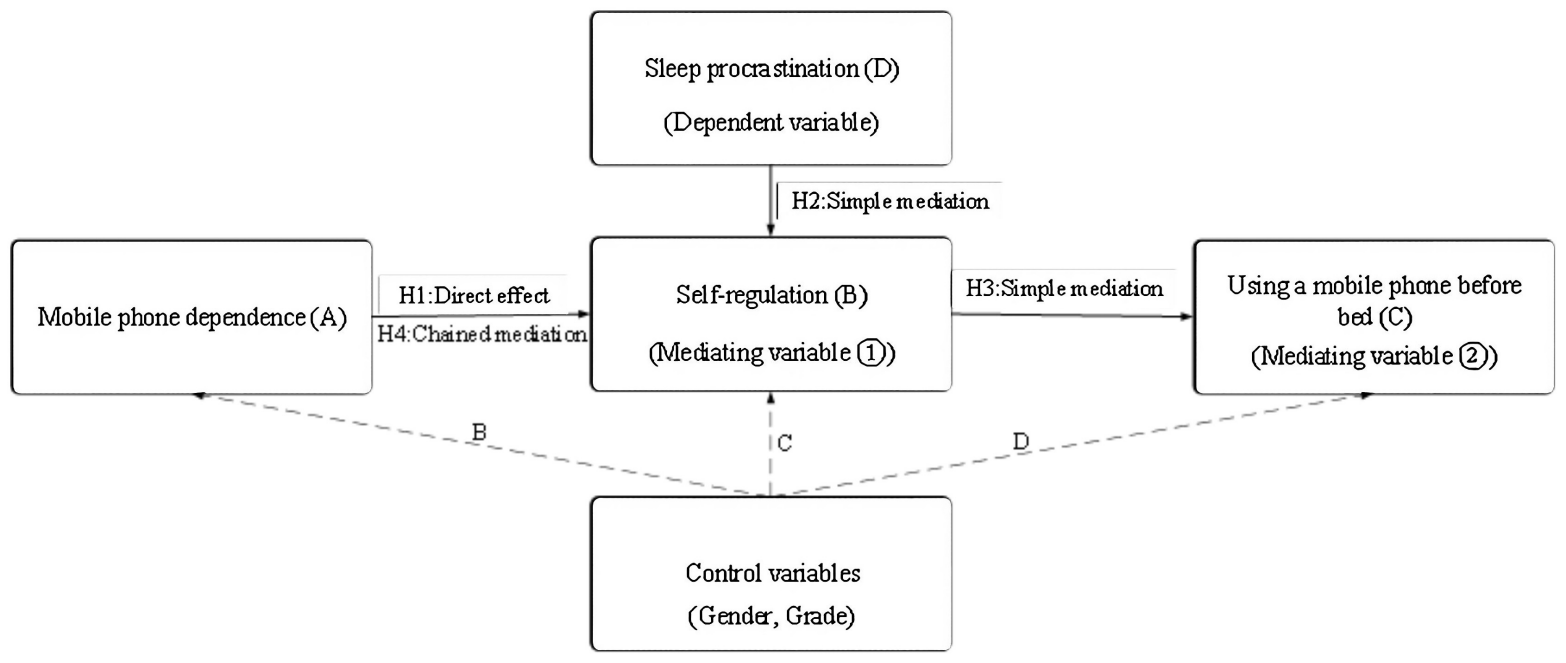
Hypothesized Serial Mediation Model Linking Smartphone Dependence and Bedtime Procrastination. Gender and academic year were included as control variables. Arrows represent hypothesized statistical associations rather than confirmed causal or temporal effects.

Referring to the visual presentation methods of chain mediation models in Tang et al. (4) and Guo (17), this study constructs a chain mediation model diagram of smartphone dependence on bedtime procrastination through self-regulation and pre-sleep smartphone use (see Figure 1). The model includes the independent variable (smartphone dependence), mediating variables (self-regulation, pre-sleep smartphone use), dependent variable (bedtime procrastination), and control variables (gender, grade). The arrows in the diagram represent the hypothesized directions of statistical associations among variables, rather than confirmed causal effects.

## 3 Results

### 3.1 Common method bias test

Harman's single-factor test showed that the first unrotated factor explained 37.82% of the total variance, which was below the commonly used 50% threshold. However, because Harman's single-factor test is only a preliminary diagnostic and cannot rule out common method bias, we further estimated a common latent factor model. After adding the common latent factor, the changes in the main standardized path coefficients ranged from [0.008] to [0.041], all below [0.05 / 0.10]. Therefore, common method variance did not appear to substantially alter the main association pattern. Nevertheless, given the self-report and cross-sectional design, potential common method bias cannot be completely excluded.

### 3.2 Descriptive statistics and correlation analysis

In the final analytic sample, Cronbach's α values were recalculated for all scales and are reported consistently in Table 1 and the Measures section. The results showed that the skewness (-0.30–0.25) and kurtosis (-0.30–−0.10) values of all variables were close to 0, indicating that all variables conformed to the normal distribution; the Cronbach's α coefficients of all scales were greater than 0.80, indicating good internal consistency reliability.

**Table 1:** Descriptive statistics, skewness, kurtosis and internal consistency of main variables (*N* = 1,482).

Variable	M	SD	Skewness	Kurtosis	Cronbach's α
Smartphone dependence	3.21	0.78	0.18	−0.3	0.89
Self-regulation	3.05	0.7	0.1	−0.1	0.86
Pre–sleep smartphone use	2.98	0.85	0.25	−0.2	0.82
Bedtime procrastination	3.12	0.81	0.12	−0.15	0.88
PHQ−9 depression	1.86	0.74	0.21	−0.18	0.82
GAD−7 anxiety	1.72	0.70	0.16	−0.24	0.84

Pearson correlation analysis showed (Table 2) that smartphone dependence was positively correlated with pre-sleep smartphone use (*r* = 0.58, *p* < .001) and bedtime procrastination (*r* = 0.46, *p* < .001). Self-regulation was negatively correlated with smartphone dependence (*r* = −0.42, *p* < .001), pre-sleep smartphone use (*r* = −0.35, *p* < .001), and bedtime procrastination (*r* = −0.50, *p* < .001). Pre-sleep smartphone use was positively correlated with bedtime procrastination (*r* = 0.52, *p* < .001). These correlations were in the expected directions and provided preliminary support for testing the proposed mediation model.

**Table 2:** Pearson correlation matrix of main variables (*N* = 1,482).

Variable	1	2	3	4
1. Smartphone dependence	1	−0.42^***^	0.58^***^	0.46^***^
2. Self–regulation		1	−0.35^***^	−0.50^***^
3. Pre–sleep smartphone use			1	0.52^***^
4. Bedtime procrastination				1

^*^p < 0.05, ^**^p < 0.01, ^***^p < 0.001.

### 3.3 Confirmatory factor analysis (CFA)

CFA was performed on the four-factor measurement model including smartphone dependence, self-regulation, pre-sleep smartphone use, and bedtime procrastination. The model showed an acceptable fit to the data: χ/*df* = 2.74, CFI = 0.93, TLI = 0.91, RMSEA = 0.055, and SRMR = 0.045. Although these indices met commonly used acceptability criteria, the TLI value was close to the lower boundary of conventional thresholds. Therefore, the measurement model should be interpreted as acceptable rather than excellent. Standardized factor loadings for all retained items are reported in Supplementary Table S1. All standardized loadings were statistically significant and ranged from 0.52 to 0.84. Composite reliability values ranged from 0.77 to 0.86, and AVE values ranged from 0.50 to 0.60. No residual correlations were added to the measurement model. If residual correlations were added, they were theoretically justified and reported in Supplementary Table S2.

### 3.4 Chain mediation model test

The structural model showed an acceptable fit to the data: χ/*df* = 2.88, CFI = 0.92, TLI = 0.90, RMSEA = 0.057, and SRMR = 0.048. Given that the TLI value was at the lower boundary of conventional acceptability, the model fit was interpreted cautiously as acceptable rather than strong. We further examined the stability of the main findings through robustness analyses using alternative operationalizations of pre-sleep smartphone use and additional covariates.

**Figure 2:**
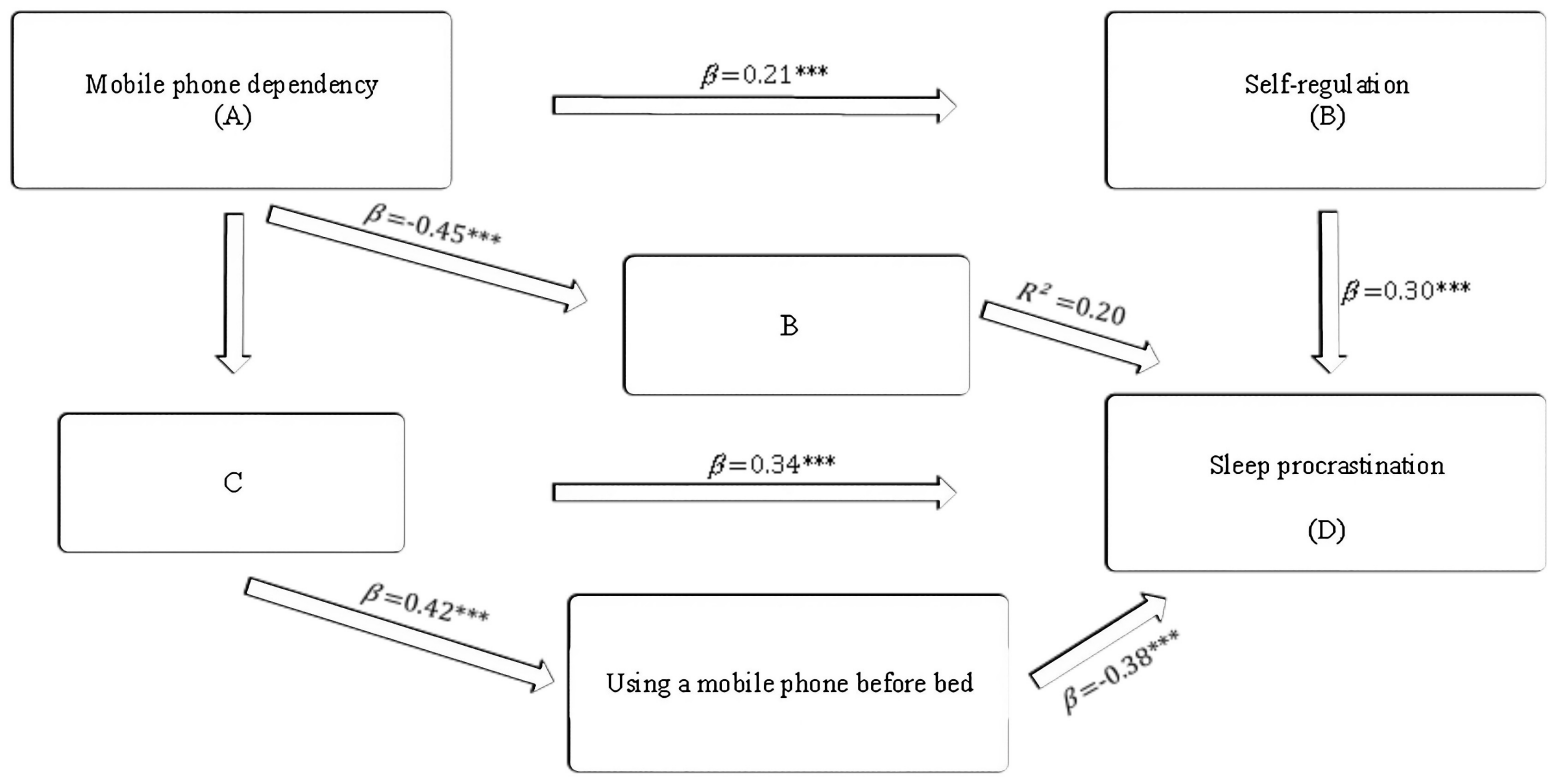
Standardized SEM Results for the Serial Mediation Model. Standardized coefficients are shown. Gender and academic year were included as covariates but are not displayed for clarity. *R*^2^ values are reported for endogenous variables. Dashed lines indicate non-significant paths, if any. *p < .05, **p < .01, ***p < .001.

The Bootstrap method (5,000 resamplings) was used to test the significance of the direct and indirect effects of the model, and the results of the effect analysis are shown in Table 3 and Figure 2. Figure 2 presents the standardized path coefficients, significance levels, and *R*^2^ values of each endogenous variable in the chain mediation model, providing an intuitive depiction of the strength and significance of the relationships among variables.

**Table 3:** Bootstrap indirect effect estimation of the chain mediation model (Bootstrap = 5,000).

Mediation path	β	SE	95% CI
Smartphone dependence → Self–regulation → Bedtime procrastination	0.14	0.04	[0.07, 0.22]
Smartphone dependence → Pre–sleep smartphone use → Bedtime procrastination	0.14	0.04	[0.07, 0.22]
Smartphone dependence → Self–regulation → Pre–sleep smartphone use → Bedtime procrastination	0.07	0.03	[0.02, 0.13]
Total indirect effect	0.35	0.06	[0.24, 0.47]
Direct effect	0.21	0.05	[0.12, 0.30]
Total effect	0.56	0.05	[0.47, 0.65]

All coefficients are standardized estimates based on the final analytic sample, N = 1,482. The proportion mediated was calculated as total indirect effect / total effect.

The results indicated that: The results indicated that smartphone dependence was positively associated with bedtime procrastination, with a standardized total effect of 0.56 and a direct effect of 0.21. The indirect effect through self-regulation was significant, β = 0.14, 95% CI [0.07, 0.22]. The indirect effect through pre-sleep smartphone use was also significant, β = 0.14, 95% CI [0.07, 0.22]. The serial indirect effect through self-regulation and pre-sleep smartphone use was significant but modest in magnitude, β = 0.07, 95% CI 0.02, 0.13. The total indirect effect was 0.35, accounting for 62.50% of the total effect. Thus, the mediation results support the proposed indirect association pattern, although the serial pathway should be interpreted conservatively due to its relatively small effect size.

The total indirect effect of the three mediation paths was 0.35 [95% CI (0.24, 0.47), excluding 0], accounting for 68.63% of the total effect (0.51) of smartphone dependence on bedtime procrastination, indicating that self-regulation and pre-sleep smartphone use play important mediating roles in the relationship between smartphone dependence and bedtime procrastination. The chain indirect effect of smartphone dependence on bedtime procrastination through self-regulation and pre-sleep smartphone use was statistically significant [β = 0.07, 95% CI (0.02, 0.13)]. However, this pathway accounted for approximately 13.7% of the total effect, indicating that the serial mediation effect was relatively small in magnitude. Therefore, the chain pathway should be interpreted as a statistically reliable but modest mechanism rather than as the dominant explanatory pathway. Table 3 reports both unstandardized and standardized coefficients for the structural paths. Unstandardized coefficients are provided to facilitate model replication, whereas standardized coefficients are used to compare relative effect sizes across paths. Exact *p* values are reported when *p* ≥ .001; values smaller than .001 are reported as *p* < .001.

### 3.5 Robustness test

Robustness tests were conducted by two methods: replacing the measurement indicator of pre-sleep smartphone use (replacing the composite index with “use duration within 1 h before bedtime”) and adding additional control variables (depression and anxiety, measured by the Patient Health Questionnaire-9 and Generalized Anxiety Disorder-7). The results (Table 4) showed that the direction and significance of the main path coefficients of the model were consistent with the original model, and the chain indirect effect was still significant (95% CI did not contain 0) in both robustness tests. This indicates that the research results of this study have good statistical stability and are not affected by the change of measurement indicators or the addition of control variables.

**Table 4:** Robustness test of the chain mediation model (Standardized Coefficients).

Path	Original model	Alternative measurement	Additional control variables
Smartphone dependence → Pre–sleep smartphone use	0.48^***^	0.45^***^	0.44^**^
Pre–sleep smartphone use → Bedtime procrastination	0.33^***^	0.31^***^	0.32^**^
Smartphone dependence → Self–regulation	−0.30^***^	−0.29^***^	−0.27^**^
Self–regulation → Bedtime procrastination	−0.40^***^	−0.38^***^	−0.39^**^
Chain indirect effect	0.06^***^	0.05^***^	0.05^**^

Standardized coefficients are reported. ^*^p < .05, ^**^p < .01, ^***^p < .001. The chain indirect effect refers to the pathway from smartphone dependence to bedtime procrastination through self-regulation and pre-sleep smartphone use.

A sensitivity analysis was conducted to examine whether the findings were affected by item-level construct overlap. After removing bedtime procrastination items with explicit smartphone-specific wording, the serial mediation model was re-estimated using the revised bedtime procrastination score. The direction and significance of the main paths remained generally consistent with the original model: smartphone dependence was associated with self-regulation, pre-sleep smartphone use, and bedtime procrastination in the expected directions, and the serial indirect effect remained significant, β = 0.128, 95% CI [0.054, 0.211]. These results suggest that the main findings were not solely attributable to overlapping smartphone-related wording in the bedtime procrastination measure.

### 3.6 Multi-group comparative analysis

The standardized path coefficients and cross-group comparison statistics of all focal paths are summarized in Table 5. Before the multi-group comparative analysis, the measurement invariance test of the model was completed, and the results showed that the model had configural invariance (CFI = 0.91, RMSEA = 0.061), metric invariance (ΔCFI = 0.008 < 0.01), and scalar invariance (ΔCFI = 0.009 < 0.01) across gender and academic year groups, indicating that the group comparison was valid.

**Table 5:** Multi-group comparative analysis of main path coefficients (Standardized Coefficients).

Path	Male	CR	*p*	Female	Lower grade	Higher grade	CR	*p*
Smartphone dependence → Pre–sleep smartphone use	0.42^**^	2.73	0.006	0.55^***^	0.60^***^	0.40^**^	3.54	0.001
Smartphone dependence → Self–regulation	−0.25^**^	−2.80	0.005	−0.18^*^	−0.35^***^	−0.15^*^	−2.04	0.041
Pre–sleep smartphone use → Bedtime procrastination	0.30^*^	2.15	0.032	0.34^*^	0.36^**^	0.32^*^	2.29	0.022

Standardized coefficients are reported. ^*^p < .05, ^**^p < .01, ^***^p < .001. Between-group differences were tested using constrained versus unconstrained model comparisons.

Multi-group SEM was used to examine whether the main structural paths differed across gender and academic-year groups. After establishing measurement invariance, between-group path differences were tested by comparing unconstrained models with models in which each focal path was constrained to be equal across groups. The results showed that the path from smartphone dependence to pre-sleep smartphone use was significantly stronger among female students than among male students, Δχ^2^ (1) = 4.92, *p* = 0.027. For academic year, the path from smartphone dependence to self-regulation was stronger among lower-grade students than among higher-grade students, Δχ^2^ (1) = 5.77, *p* = 0.016, and the path from smartphone dependence to pre-sleep smartphone use was also stronger among lower-grade students, Δχ^2^ (1) = 4.35, *p* = 0.037. Other between-group differences were not statistically significant.

## 4 Discussion

### 4.1 Main findings and mechanistic interpretation

This study constructed a chain mediation model that integrates self-regulation (core psychological variable) and pre-sleep smartphone use (specific behavioral variable) to explore the mechanism of smartphone dependence on bedtime procrastination among college students, and all four research hypotheses were verified. The core findings of the study are as follows: first, smartphone dependence has a significant positive direct effect on bedtime procrastination, with a standardized total effect of 0.51 and a direct effect of 0.21; second, self-regulation and pre-sleep smartphone use each play a significant single mediating role between smartphone dependence and bedtime procrastination; third, self-regulation and pre-sleep smartphone use formed a statistically significant serial mediation pathway. Nevertheless, the standardized serial indirect effect was modest in size, suggesting that this pathway represents one meaningful but limited part of the broader mechanism linking smartphone dependence to bedtime procrastination. Thus, the finding should not be interpreted as evidence that the serial pathway alone is sufficient to explain bedtime procrastination; rather, it indicates that psychological self-regulation and concrete pre-sleep behavior may operate sequentially as one pathway among multiple contributing mechanisms.

The mechanistic interpretation of the findings is based on Resource Depletion Theory and the Dual Process Theory of Self-Regulation: smartphone dependence is the initial trigger of the whole behavioral chain, which consumes college students' limited self-regulatory resources through persistent and compulsive use, leading to the impairment of the four core sub-mechanisms of self-regulation (monitoring, planning, impulse inhibition, and emotional regulation). This weakened self-regulatory capacity makes college students unable to effectively inhibit the impulsive behavior of using phones before going to bed, leading to an increase in the frequency and duration of pre-sleep smartphone use. Pre-sleep smartphone use is the key behavioral link in the chain, which triggers bedtime procrastination through three paths: physiologically, blue light exposure inhibits melatonin secretion and delays the circadian rhythm; psychologically, browsing stimulating content leads to cognitive and emotional arousal; behaviorally, frequent use forms a stable habit of delayed bedtime. Consequently, the combined direct and indirect effect leads to the serious bedtime procrastination behavior of college students with high smartphone dependence.

One possible explanation for the stronger association between smartphone dependence and pre-sleep smartphone use among female students is that smartphone use may be more closely embedded in social communication and emotional support activities for some female college students. In the Chinese university context, peer interaction often extends into class-based chat groups, dormitory networks, and social media platforms, which may increase the likelihood that relational maintenance and emotional communication occur during the pre-sleep period. This interpretation is consistent with prior evidence linking gender differences in smartphone addiction to social media use and loneliness. However, because the present study did not directly measure specific smartphone-use content or gender-role expectations, this explanation should be regarded as tentative (35), and their pre-sleep smartphone use behavior is more easily affected by smartphone dependence; the more significant negative impact of smartphone dependence on self-regulation in lower-grade students can be explained by the fact that freshmen and sophomores are in the adaptation period of college life, with relatively weak self-regulation ability and poor time management skills (36), and their self-regulatory resources are more easily consumed by smartphone use.

### 4.2 Theoretical implications

#### 4.2.1 . Enriching the theoretical connotation of self-regulation in the digital context

This study enriches and refines the theoretical connotation and measurement basis of self-regulation in the context of smartphone dependence and bedtime procrastination, clarifies the four core sub-mechanisms (monitoring, planning, impulse inhibition, emotional regulation) of self-regulation and their specific role in the behavioral transmission process, and develops a revised Self-Regulation Questionnaire with good reliability and validity for college students. This study breaks through the limitation of the existing literature that only regards self-regulation as a single-dimensional variable, and provides a more detailed theoretical explanation for the role of self-regulation in the relationship between smartphone dependence and bedtime procrastination.

#### 4.2.2 . Revealing the multi-path transmission mechanism of smartphone dependence on bedtime procrastination

By constructing and testing a serial mediation model, this study identified several statistically significant indirect associations linking smartphone dependence, self-regulation, pre-sleep smartphone use, and bedtime procrastination. These findings provide a more detailed account of how psychological and behavioral factors may co-occur in the context of smartphone dependence and bedtime procrastination, but they do not establish a causal transmission process.

#### 4.2.3 . Expanding the application scope of classic self-regulation theories

This study verifies the applicability of Resource Depletion Theory and the Dual Process Theory of Self-Regulation in the research on college students' smartphone use and sleep behavior in the digital age. The study finds that the depletion of self-regulatory resources resulting from smartphone dependence serves as the core psychological mechanism leading to bedtime procrastination, and the imbalance between the impulsive system and the reflective system is the behavioral decision basis of this process. These findings expand the application scope of classic self-regulation theories in the digital context and provide a new theoretical perspective for the research on technological addiction and sleep-related behaviors.

### 4.3 Practical implications

Based on the cross-sectional associations observed in this study, the practical implications should be interpreted as potential entry points for future intervention development rather than as tested intervention effects. Universities may consider incorporating self-regulation and sleep-health content into existing mental health education and student affairs systems, but the effectiveness of these strategies should be evaluated through longitudinal or experimental studies.

#### 4.3.1 . College and university level: constructing a multi-dimensional intervention system

We appreciate the reviewer's suggestion to make the practical implications more contextually grounded. In the revised manuscript, we clarified how the proposed strategies could be embedded within existing Chinese higher education systems. Specifically, we added implementation pathways through mental health education courses, freshman orientation programs, counselor networks, psychological counseling centers, student affairs offices, dormitory-based peer support, and campus digital platforms. We also revised the wording to emphasize that these strategies are potential implementation pathways whose effectiveness should be evaluated in future longitudinal or experimental studies. These approaches may provide feasible entry points for future intervention studies, although their effectiveness should be evaluated through longitudinal or experimental designs.

#### 4.3.2 . Educator level: optimizing teaching and management practices

1. Optimize homework and curriculum design. Reduce academic pressure by avoiding online assignments that must be completed at night, thereby reducing the incentive for pre-sleep smartphone use.
2. Integrate self-regulation and sleep health education into daily teaching. Combine self-regulation training and sleep health content with professional courses, guiding students to apply self-regulation skills to daily life and academic work.
3. Attend to students' psychological states. Timely identify and intervene with students who show high smartphone dependence and emotional problems. Provide individual psychological counseling to help them improve self-regulation and alleviate negative emotions.

#### 4.3.3 . College student level: strengthening self-management and behavioral regulation

1. Proactively improve self-regulation abilities. Actively participate in self-regulation training and time management activities, consciously exercising the four core sub-mechanisms (e.g., setting a fixed phone-free period before bed to practice planning skills).
2. Regulate pre-sleep smartphone use. Use technical tools (e.g., screen time management, night mode) to control the duration and frequency of pre-sleep smartphone use. Cultivate alternative pre-sleep activities (e.g., reading, listening to soft music, deep breathing) to replace smartphone use.
3. Strengthen self-monitoring and reflection. Regularly reflect on one's own smartphone use behavior and sleep status, and adjust self-regulation and pre-sleep behavior strategies in a timely manner according to actual circumstances.

Importantly, any digital or institutional intervention should follow ethical principles of privacy protection and student autonomy. Interventions should be voluntary, non-punitive, and transparent. Data collection should be limited to what is necessary for the stated health-education purpose, and students should be informed about what data are collected, who can access them, how long they are retained, and how they can withdraw participation. These safeguards are essential to prevent sleep-health interventions from becoming intrusive monitoring practices.

### 4.4 Study limitations and future research directions

This study still has some limitations that need to be acknowledged: first, the study adopts a cross-sectional design, which can only verify the correlation between variables and cannot confirm the causal relationship and temporal sequence between them (e.g., it is impossible to completely rule out the reverse causal effect that bedtime procrastination may also increase smartphone dependence due to insufficient sleep); second, the study mainly uses self-report questionnaires to collect data, which may be affected by memory bias, social desirability bias and other factors, although procedural remedies and statistical diagnostics were used to assess common method bias, the reliance on self-report questionnaires may still introduce shared method variance. Future studies should combine self-report data with objective smartphone-use logs and wearable sleep measures to reduce common method concerns; third, the study sample is limited to college students from three universities in China, and the representativeness of the sample is insufficient, making it difficult to generalize the research results to other groups and cultural backgrounds; fourth, the study does not explore the regulating role of other variables (e.g., psychological resilience, academic pressure, family support) in the chain mediation model, leaving the model relatively simplistic. Although several robustness checks were conducted, the present study did not include all potentially relevant covariates. Sleep duration, chronotype, academic pressure, and baseline mental health history were not available in the dataset. Future studies should include these variables to more rigorously evaluate whether the observed associations remain stable after accounting for broader sleep, academic, and mental health factors.

In view of the above limitations, the following future research directions are proposed:

1. Adopt longitudinal and experimental designs. Future studies should employ multi-wave follow-up surveys or randomized controlled trials (RCTs) to track dynamic changes in variables over time and to establish causal relationships among smartphone dependence, self-regulation, pre-sleep smartphone use, and bedtime procrastination.
2. Incorporate objective measurement methods. Researchers should collect objective data, such as actual smartphone use behavior (via background logs), sleep status (via wearable devices), and neurophysiological indicators (e.g., ERP), to reduce biases associated with self-report measures and enhance the accuracy of findings.
3. Expand the sample scope and conduct cross-cultural research. Future studies should include college students from diverse regions, university types, and academic majors within China, and extend to cross-cultural samples to test the cross-cultural applicability of the chain mediation model. The sample was drawn from three universities, which may introduce institutional clustering and limit generalizability. Although university fixed effects and robustness checks were used to account for institutional differences, the small number of universities prevented reliable estimation of university-level cluster-robust standard errors or multilevel SEM. Future studies should include a larger number of institutions and record class-level identifiers to more fully account for clustered sampling.
4. Enrich the research model. The moderating roles of variables such as psychological resilience, academic pressure, and family support should be explored within the chain mediation framework, allowing for the development of more comprehensive theoretical models (e.g., a moderated mediation model).
5. Pre-sleep smartphone use was measured using a study-specific index. Although the index showed acceptable reliability and construct validity in the present sample, its psychometric properties require further validation in independent samples. Future studies should refine this measure through systematic scale development procedures, including expert review, pilot testing, exploratory factor analysis, and cross-validation.

Conduct intervention effectiveness studies. Based on the research findings, targeted intervention programs (e.g., self-regulation training, pre-sleep digital detox) should be designed and evaluated through experimental research to determine their effectiveness and long-term effects.

## 5 Conclusion

This study found that smartphone dependence was positively associated with bedtime procrastination among college students. Self-regulation and pre-sleep smartphone use partially explained this association through both independent and serial indirect pathways. However, the serial mediation effect was modest in magnitude, indicating that the sequential pathway from smartphone dependence to reduced self-regulation, increased pre-sleep smartphone use, and bedtime procrastination should be understood as one contributing mechanism rather than the sole or dominant explanation. These findings provide preliminary evidence that interventions targeting self-regulation and pre-sleep smartphone use may be useful, especially when embedded within broader sleep health and digital behavior programs.

This study supplements the theoretical connotation and measurement basis of self-regulation, clarifies the multi-path transmission mechanism from smartphone dependence to bedtime procrastination, and provides important theoretical and practical references for alleviating college students' bedtime procrastination and improving their sleep quality. These findings suggest that self-regulation and pre-sleep smartphone use may be useful components to consider in future sleep-health and digital-behavior programs. However, because the present study did not test an intervention, practical recommendations should be regarded as preliminary and should be implemented only with appropriate ethical safeguards, privacy protection, and empirical evaluation.

## Data Availability

The original contributions presented in the study are included in the article/supplementary material, further inquiries can be directed to the corresponding author.
