# Supplementary material for: The chain mediation effect of self-regulation and pre-sleep smartphone use on the relationship between smartphone dependence and bedtime procrastination among college students
Source: Front Public Health. 2026 Aug 4;14:1894257. doi: 10.3389/fpubh.2026.1894257 (PMC13482389; doi:10.3389/fpubh.2026.1894257)
Supplement: Supplementary file 1 [file Table_1.docx]

**Table S1**. Supplementary Parameter Table

| **Construct** | **Item code** | **Item wording** | **Response scale** | **Standardized loading** |
| --- | --- | --- | --- | --- |
| Smartphone dependence | SD1 | ... | 1–5 | [0.79] |
| Self-regulation | SR1 | ... | 1–5 | [0.74] |
| Pre-sleep smartphone use | PSU1 | ... | 1–5 | [0.71] |
| Bedtime procrastination | BP1 | ... | 1–5 | [0.76] |
